# Endoscopic direct-vision appendicitis therapy for the treatment of chronic appendicitis with multiple appendicoliths

**DOI:** 10.1055/a-2602-3154

**Published:** 2025-06-18

**Authors:** Li-Hua Ren, Yuan Ding, Yuanyuan Li, Yadong Feng, Lin Yang, Yuan-Yuan Han, Rui-Hua Shi

**Affiliations:** 1 Department of Gastroenterology, Zhongda Hospital, Southeast University, Nanjing, China; 2 12579 School of Medicine, Southeast University, Nanjing, China; 3 572403 School of Public Health, Soochow University, Suzhou, China; 4 12461 Nanjing Medical University, Nanjing, China

Appendicoliths are considered as a well-established etiology factor in both acute appendicitis and chronic abdominal pain syndromes 1 . While appendectomy remains definitive for acute appendicitis, emerging endoscopic strategies, especially natural orifice transluminal endoscopic surgery (NOTES) illuminate organ-preserving alternatives, particularly in complex cases where conventional approaches falter 2 . Endoscopic retrograde appendicitis therapy (ERAT) is increasingly utilized in acute appendicitis therapy 3 4 . However, it technically depends on fluoroscopic guidance and carries risks of perforation during blind cannulation of the appendiceal lumen with contrast catheters 5 . Herein we present a case of multiple large appendicoliths with chronic obstruction successfully resolved through endoscopic direct-vision appendicitis therapy (EDAT) ( Video 1 ).

EDAT for the management of multiple appendicoliths with chronic pain in a 28-year-old man. Abbreviation: EDAT, endoscopic direct-vision appendicitis therapy.Video 1

A 28-year-old man presenting with a 1-year history of intermittent right lower quadrant dull pain, acutely exacerbated in the preceding week was admitted to our medical team. Contrast-enhanced computed tomography (CT) revealed four calculi within the appendiceal lumen with the largest measuring 1.4 cm in maximal diameter ( Fig. 1 **a–c** ). The patient declined surgical intervention and had undergone conservative medical treatment with limited efficacy. We performed EDAT ( Fig. 2 **a–i** ), which enabled complete appendicolith extraction and resulted in immediate resolution of abdominal pain. Intravenous antibiotics were administered for 24 hours, and a semi-liquid diet was initiated on postoperative day 2 (POD 2). The patient was discharged on POD 3 without recurrence of symptoms.

**Fig. 1:**
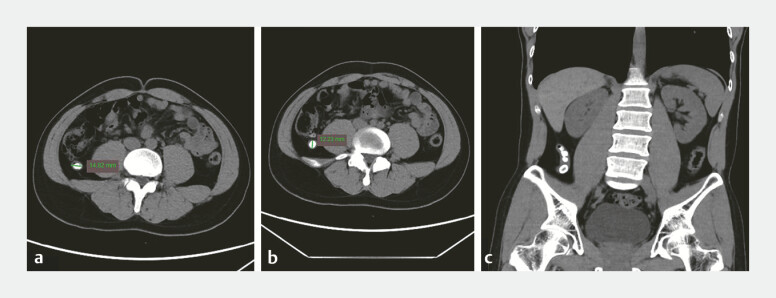
CT imagings prior to EDAT. **a** A calcified stone with a maximum axial diameter of 14.82 mm. **b** Another stone exhibiting a maximal longitudinal dimension of 12.23 mm. **c** Coronal reconstruction revealing clustered appendicoliths. Abbreviations: CT, computed tomography; EDAT, endoscopic direct-vision appendicitis therapy.

**Fig. 2:**
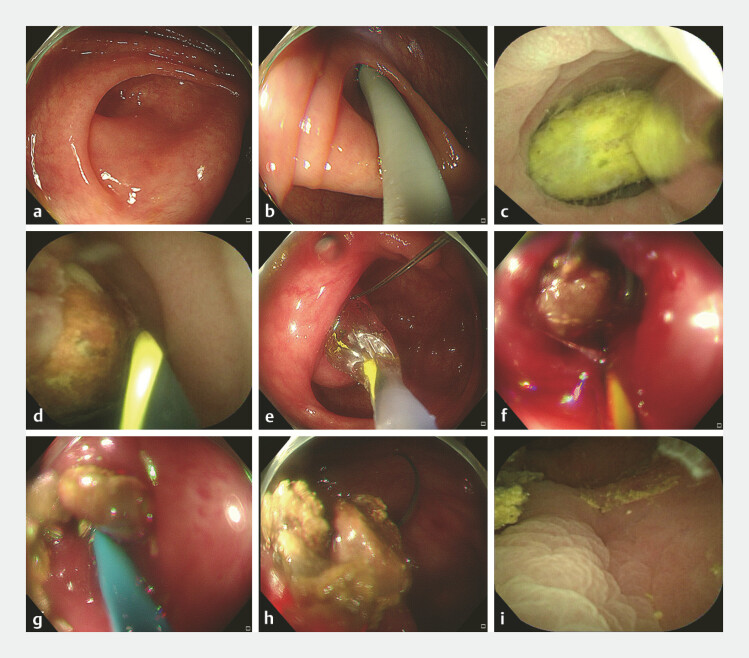
EDAT for the appendicolith management. **a** A colonoscope was advanced to the ileocecal region, and identified the appendiceal orifice. **b** A choledochoscope was accessed through the biopsy channel and angled leftward and upward into the appendiceal lumen under direct visualization. **c** Multiple appendicoliths were visualized in the proximal lumen. **d** A spiral stone extraction basket was entrapped due to luminal stenosis and stone adhesion. **e** A controlled radial expansion balloon was deployed over a guidewire to dilate the stenotic segment. **f** The entrapped basket was disengaged following the appendiceal orifice dilation. **g** Subsequent calculi extraction was performed using a balloon-anchored traction. **h** Multiple stones were extracted into the colonic lumen. **i** Post-procedural choledochoscopic evaluation confirmed complete clearance without mucosal injury. Abbreviation: EDAT, endoscopic direct-vision appendicitis therapy.

Endoscopy_UCTN_Code_TTT_1AQ_2AH

## Notes

Endoscopy E-Videos https://eref.thieme.de/e-videos
*E-Videos* is an open access online section of the journal *Endoscopy* , reporting on interesting cases and new techniques in gastroenterological endoscopy. All papers include a high-quality video and are published with a Creative Commons CC-BY license. Endoscopy E-Videos qualify for HINARI discounts and waivers and eligibility is automatically checked during the submission process. We grant 100% waivers to articles whose corresponding authors are based in Group A countries and 50% waivers to those who are based in Group B countries as classified by Research4Life (see: https://www.research4life.org/access/eligibility/ ). This section has its own submission website at https://mc.manuscriptcentral.com/e-videos .
